# Early pathological changes of peri-coronal tissue in the *distal area* of erupted or partially impacted lower third molars

**DOI:** 10.1186/s12903-023-03082-z

**Published:** 2023-06-12

**Authors:** Dardo Menditti, Pierluigi Mariani, Diana Russo, Barbara Rinaldi, Luca Fiorillo, Marco Cicciù, Luigi Laino

**Affiliations:** 1 Multidisciplinary Department of Medical-Surgical and Odontostomatological Specialties, University of Campania “Luigi Vanvitelli”, 80121 Naples, Italy; 2 Department of Experimental Medicine, Section of Pharmacology, University of Campania Luigi Vanvitelli, Piazza Luigi Miraglia 2, 80138 Naples, Italy; 3 Department of Biomedical and Dental Sciences and Morphofunctional Imaging, School of Dentistry, University of Messina, Via Consolare Valeria, 1, 98125 Messina, Italy; 4 Department of Public Health Dentistry, Dr D.Y. Patil Dental College and Hospital, Dr D.Y. Patil Vidyapeeth, Pimpri, Pune, 411018 India; 5 Department of Surgery and Surgical Specialties, University of Catania, Catania, 95100 Italy

**Keywords:** Third molars, Peri-coronal tissues, Pathologic peri-coronal radiolucency, Dental follicle, Histological examination

## Abstract

**Aim:**

This study was performed to histologically evaluate peri-coronal tissues of partially impacted and erupted third molars that did not exhibit pathologic peri-coronal radiolucency.

**Materials and methods:**

Healthy patients with erupted or partially erupted (with part or all of the dental crown present in the oral cavity) mandibular third molars (classified as IA and IIA according to the Pell and Gregory classification) and vertically positioned (according to the Winter classification or erupted third molars) associated with peri coronal radiolucency of equal to or less than 2.5 mm. Associated with third molar surgery, tissue sampling from the distal area was performed, which was subjected to an anatomopathological examination to determine the histological nature.

**Results:**

One hundred teeth (100 patients) were selected, and 100 specimens were analyzed. 53% of the sample were included in the non-pathological group and 47% showed pathological changes (fibrotic tissue (n 15), periodontal cyst-like (n 9), squamous epithelial metaplasia (4 cases), islands of odontogenic epithelial residues organized micro-cyst with keratocystic/ameloblastic appearance (4 cases), granulation tissue (n 8), giant cell tumour (n 4) and lobular capillary hemangioma (n 4)). Pathological changes did not have differences in incidence between the gender (*p* value = 0.85) and did not show any correlation with age, (*p* value = 0,96).

**Conclusions:**

These findings suggest that radiographic appearance may not be a reliable indicator of the absence of disease within a dental follicle. Therefore, clinicians should pay attention to or follow up on even peri-coronal radiolucency of less than 2.5 mm.

## Background

Dental follicle (DF), derived from odontogenic ectomesenchyme, comprises one of the components of tooth germs. Histologically, DF is referred to as a condensed ectomesenchyme, which delimits the dental papilla and encapsulates the dental organ. It is characterized by fibrous connective tissue with reduced enamel epithelium, odontogenic epithelial rests, and myxoid tissue [1]. DF has a critical role in tooth eruption: it enlarges toward the occlusal plane, following the gubernacular canal, and leading to bone resorption, creating an eruption pathway [2]. Physiologically, when dental roots are formed, the alveolar bone increases in height and the tooth erupts; in this stage, dental follicle cells undergo apotheosis after differentiating into cementum, alveolar bone, and periodontal ligament cells [3]. Occasionally DF could remain adjacent to the crown of unerupted and impacted; teeth most frequently found in this condition are the third mandibular molars (90% of impacted teeth) [4].

Many histological variations may exist in the follicle tissue surrounding impacted teeth, including pathological changes in epithelial rests. The potential transformation into cysts (e.g. dentigerous cyst and odontogenic keratocyst) or odontogenic tumors (e.g. ameloblastoma) is related to the epithelial residues of the DF located in its connective wall, in particular the reduced enamel epithelium and remnants of dental lamina [5, 6]. Less frequently, other pathological entities have been described, including calcifying epithelial odontogenic cysts, odontogenic myxoma, odontogenic fibroma, and even odontogenic carcinomas [2, 7].

Radiographically, the DF of the impacted teeth presents as a slight semicircular radiolucency around the crowns of unerupted teeth. It is often assumed that peri coronal radiolucency of “follicular space” of size less than 2.5 mm in width is considered radiographically non-pathological and if more, it is considered radiographically pathological because the latter condition is associated with a high incidence of dentigerous cyst [8, 9]. Recently, studies showed the incidence of pathological changes also in DF of impacted teeth that have peri-coronal radiolucency less than 2.5 mm [4, 10].

In partially erupted lower third molars (class IA and IIA according to Pell and Gregory classification) in vertical position (Winter classification) and sometimes in erupted third molars, DF is absent in the mesial area in correspondence with the adjacent tooth while, it is present in the distal area of lower molar (DALM) and it could remain in the soft and hard tissues. Radiographically, this area could appear as the distal peri coronal radiolucency (“crescent moon radiolucency”) corresponding to the physiological DF when the thickness is less than 2.5 mm.

There’s still little evidence regarding the histological nature of the radiolucent lesion in DALM in partially erupted or completely erupted teeth showing “crescent moon radiolucency” less than 2.5 mm, thus the present study aimed to evaluate the early pathologic changes occurring in DF of partially impacted third molars (Pell and Gregory class IA and IIA) and erupted third molars that present DALM radiolucency less than 2.5 mm.

## Materials and methods

This prospective observational study was conducted in the oral surgery department of the University of the Campania L.Vanvitelli over a period of 36 months from January 2015 to December 2018. The interventions were performed following the Helsinki Declaration and informed consent was obtained from each patient or parent before the procedure. The study protocol was approved by our institution’s ethics committee on June 12, 2005 (Second University of Naples [SUN] Internal Registry: Experimentation #914).

The selected patients were referred to the Oral surgery department for the extraction of mandibular third molars. Healthy patients, regardless of age, were included in the study who had erupted or partially erupted (part or all of the dental crown present in the oral cavity) mandibular third molars (classified as IA and IIA according to the Pell and Gregory classification) and vertically positioned (according to the Winter classification or erupted third molars) associated with a radiolucent DALM with dimensions equal to or less than 2.5 mm.

Patients with previous odontogenic osteolytic lesions of the jaw bones or with previous acute symptoms of pericoronitis were excluded from the study.

**Fig. 1:**
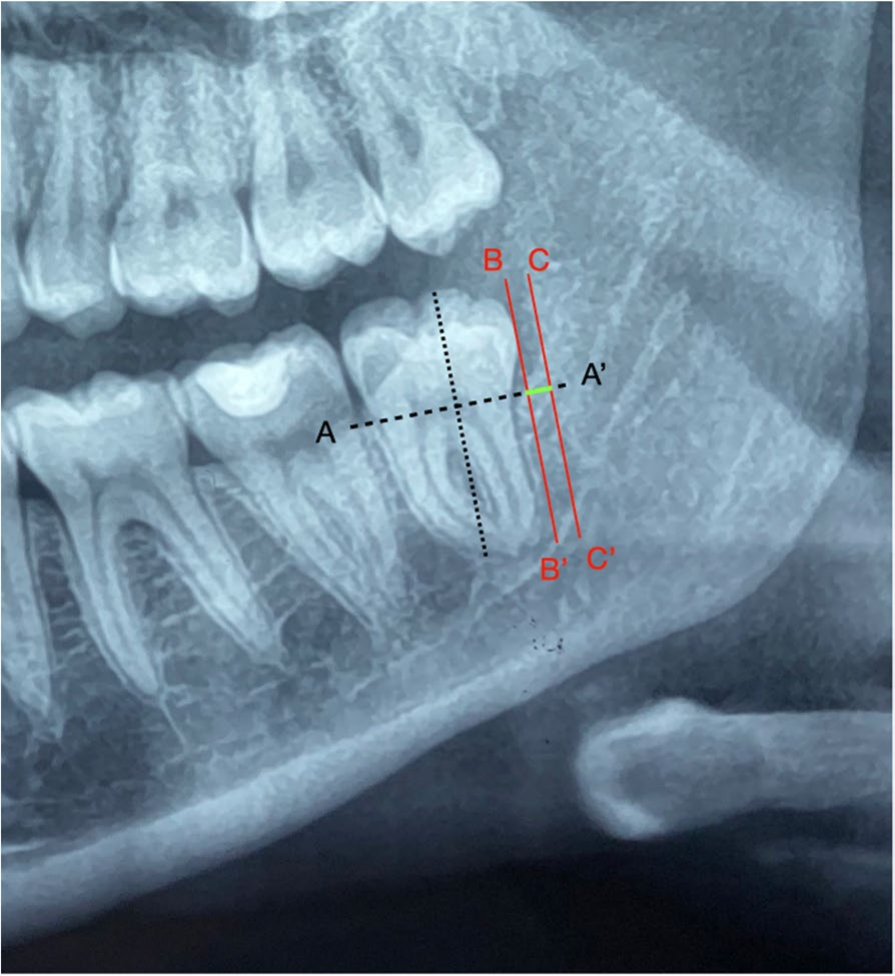
Measurement scheme on X-ray OPT for the assessment of the DALM lesion’s size

The extension of DALM radiolucency was assessed using the digital panoramic dental radiographic importing the Digital Imaging and Communications in Medicine (DICOM) data on the viewer software where the measurements were carried out following a measurement scheme (Fig. 1): two perpendicular lines were drawn on the image of the impacted teeth, of which one line passed through the cement-enamel junction (AA’) and the other line (BB’) passing through the most distal point of the crown. Another line was drawn, parallel to BB’, passing through the most distal point of the peri coronal radiolucency (CC’). The distance between BB’ and CC’ was considered the size of the DALM radiolucency.

**Fig. 2:**
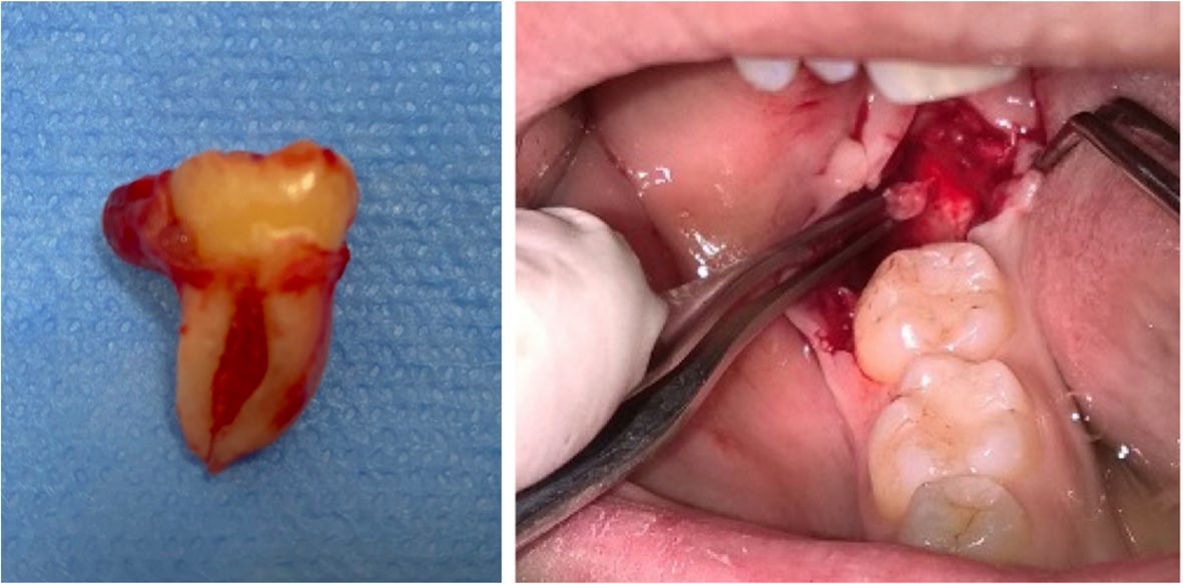
Extracted lower third molar with attached pericoronal lesion, Biopsy of DALM on the right

The surgery involved the extraction of the tooth with the associated DF (Fig. 2) or a biopsy of the DALM (Fig. 2) was performed contextually, removing the soft tissue between the bone and the overlying gingiva using scalpel n-15. All the interventions were performed under local anaesthesia and there were no complications; the patients were discharged 3 h after surgery with therapy (antibiotic and anti-inflammatory) and the removal of the suture was performed after 7 days. The specimens were sent to the Department of Pathological Anatomy of the same University for histological examination after being fixed with 10% formalin then the tissues were processed to paraffin wax. Sections (5-m thick) were cut from each block containing the DF and gingival specimens and stained with hematoxylin and eosin (H&E) for routine histologic examination. An oral pathologist, blinded to the clinical and radiological features, performed the histopathological evaluation. The samples were classified into one of two groups: non-pathological DALM and pathological DALM. The following were the histopathological criteria for the first group: i) dental follicle or its remains with or without odontogenic epithelium residues (the epithelial rest characterized by a thin epithelium formed by one to four layers of cuboidal cells and were interpreted as the reduced epithelium of the enamel organ, therefore not pathological), ii) gingival tissue with or without inflammatory infiltrate. The second group included i) findings of pathological changes of the dental follicle with cystic or neoplastic degeneration of epithelial residues (when presents squamous metaplasia in focal areas of the epithelium were interpreted as pathological changes in dental follicles), ii) other signs of pathological tissue changes of DALM including inflammatory or reactive lesions.

The Chi-squared test with Bonferroni correction was used to determine associations between the variables. The R software was used for the statistical analysis. A *p*-value < 0.05 was considered indicative of statistical significance.

## Results

In the study were included 100 patients of which 50 were males and 50 were females. The age ranged from 16 to 50 years (mean 26,8), The reason for extractions was primarily orthodontic and gnathological therapy (60 cases, 35 males and 25 females), periodontal pathologies of the second molar (23 cases) and prevention or presence of caries of the second molar (17 cases). Regarding the 100 analyzed specimens, 53 were included in the non-pathological DALM group and 47 in the pathological DALM group (Table 1); of the latter, 36% (17% of the entire sample) presented pathological alterations of epithelial origin.

**Table 1:** Distribution of histological findings of DALM

	** < 25 years old**		** > 25 years old**		TOT
**Non-pathological DALM**	**Male**	**Female**	**Male**	**Female**	
Gingival tissue with inflammatory infiltration	4	8	3	3	18
Physiological gingival tissue	3	2	3	2	10
Follicular tissue	8	9	4	4	25
**Pathological DALM**					
Parodontal like cyst	3	3	2	1	9
Granulation tissue	2	3	2	1	8
Squamous metaplasia with intraparietal micro-cyst	2	1	1	0	4
Fibrotic tissue	6	3	3	3	15
Islands of odontogenic epithelial residues with keratocystic/amelobastic appearance	1	1	1	1	4
Giant cell tumor	0	2	0	2	4
Lobular capillar hemangioma	1	1	0	1	3
TOTAL	30	33	19	18	100

**Fig. 3:**
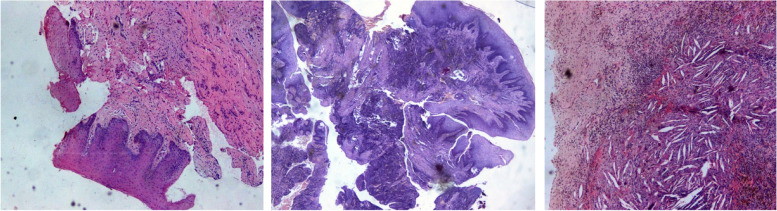
Photomicrographs show from left: multi-layered non-keratinizing squamous epithelium with focally "plaque-like" epithelial proliferations with histological appearance of periodontal cyst (hematoxylin and eosin [H&E]; original magnification × 1); Intraparietal island of epithelial cells with histological appearance of satellite micro-cyst ([H&E]; original magnification × 1); Squamous metaplasia with a cholesterol-rich wall resembling odontogenic cystic formation ([H&E]; original magnification × 10)

About the first group, the most common finding was physiological dental follicle (25 cases), followed by gingival tissue with inflammatory infiltrate (18 cases) and physiological gingival tissue (10 cases). In the second group, the most represented pathological finding was fibrotic tissue (15 cases), periodontal cyst-like (9 cases) (Fig. 3), findings suggestive of focal squamous epithelial metaplasia with intraparietal epithelial islands organized in micro-cyst (4 cases) (Fig. 3), islands of odontogenic epithelial residues organized micro-cyst with keratocystic/ameloblastic appearance (4 cases) (Fig. 3), granulation tissue (8 cases), giant cell tumour (4 cases) and lobular capillary hemangioma (pyogenic granuloma) (3 cases). Pathological changes did not have differences in incidence between the two genders (statistical analysis did not reveal any significant difference, *p* value = 0.85), and did not show any correlation with age (no significant difference between the < 25 years old group and the > 25 years old, *p*-value = 0,96).

## Discussion

The DF and included epithelial residues of unerupted teeth may have different fates, thus they could remain quiescent or be subject to pathological changes, probably caused by chronic inflammatory stimuli. Several studies showed that pathological changes, such as the development of dentigerous cysts, odontogenic keratocyst, and ameloblastoma, are eventually been diagnosed even in radiographically physiological peri coronal tissues [4, 10, 11]; this substantiates the hypotheses in favour of prophylactic removal of the impacted third molar, and it’s opposed to the current orientation to remove asymptomatic impacted teeth when the peri coronal radiolucency is greater than 2.5 mm, according to Glosser and Campbell who defined radiographic pathology was as peri coronal radiolucency of 2.5 mm or more [8].

Regarding partially erupted and erupted teeth with radiolucency in DALM, there is not enough evidence that defines the histological nature of the tissue surrounding the teeth. The purpose of this study was to investigate the histologic nature of the DALM tissue of erupted or partially impacted lower third molar when the dimension of the “crescent moon radiolucency” is less than 2.5 mm.

The results showed that almost half of the analyzed DALM samples (47%) were characterized by pathological alterations of the tissue, including early cystic/neoplastic changes of odontogenic epithelial residues, and pathological features of inflammatory and reactive origin such as granulation tissue, giant cell tumour, and lobular capillary hemangioma.

These results are partially in agreement with those present in the literature, although most of the existing studies included impacted teeth, or some of them included both impacted and partially impacted ones.

Regarding the follicular space less than 2.5 mm in impacted wisdom teeth, Baykul in a study on 94 patients reported 50% pathologic change similar to the present study; although the teeth included in this study were all impacted it is interesting to note that 75% of the impacted teeth in vertical position show pathological changes [12]. Other studies such as that of Adelsperger [13] and Glosser [14].reported a lower incidence of pathological degeneration respectively of 34% and 32%, although only squamous metaplasia or dentigerous cyst were considered as pathological changes in these studies; this value is higher than that of the present paper as in our work only 17% of the entire sample showed alterations of epithelial origin.

Furthermore, some of the previous studies reported the association between age and incidence of pathological changes underlining those patients older than the second decade showed a higher rate of pathological changes [12, 13]; these results were not found in the present study, as no significant association with age was found in our analyses.

Is noteworthy that this study includes only teeth that are partially erupted in the oral cavity, therefore not completely surrounded by the DF, thus the pathological changes reported in this study could not only be derived from the DF but could derive from all the cellular and tissue components that could constitute the DALM, in particular, periodontal tissues.

In this context, partially erupted teeth generate a distal pocket that is difficult to clean, and this supports a persistent subclinical inflammatory condition [15]. When epithelial damage occurs, fibrosis (most common pathological findings in the present study, 31.9%) and granulation tissue could develop in subepithelial tissue [16]. Reactive lesions such as peripheral giant cell tumour and lobular capillary hemangioma, although they were the least frequent findings, represented 8% and 6.38% respectively of all pathological changes occurring in DALM. The pathological change of the epithelial cells is commonly represented by squamous metaplasia which is an adaptive mechanism to chronic reversible damage when cells are constantly affected by non-lethal impulses [17], although this alteration was found in 4% of the whole sample, instead findings of cyst degeneration (like periodontal cysts with the characteristic bland stratified squamous epithelium with plaque-like thickening exhibiting a whorled morphology) or islands organized in micro-cyst were founded in 17% of the specimens.

These findings suggest that the lack of radiographic appearance of the disease is not a reliable indicator of the absence of disease, even in the case of partially erupted teeth in the oral cavity, and that the prevalence of soft tissue pathologies is higher than generally assumed from radiographic evaluation alone.

The radiographs mostly used in oral surgery are the dental panoramic, and often these are the only ones to be used in the extractive surgery of mandibular third molars for diagnosis, to evaluate the anatomic relationships of the impacted tooth, and to plan the treatment. In traditional panoramic, radiographic artefacts are generated in the opposite location of the object, and horizontal and vertical enlargement is possible with limitations to the evaluation of the anatomical [18, 19]. Moreover, this imaging cannot be considered standard: the traditional radiographic equipment for the dental panoramic, the films, and their development are not similar to each other and although digital s seem to allow sufficient standardization in radiological measurement, they can be achieved with various imaging devices that are not uniform [18, 19].

Therefore, relying only on a radiographic image and on a metric measurement to decide whether a condition can be defined as pathological could be reductive and could even lead to the misdiagnosis of initial pathological conditions that could evolve into pathologies that require more complex surgical treatment.

Due to selection bias in this type of study, it’s not possible to state the validity of prophylactic removal of asymptomatic impacted teeth to prevent the development of pathologically significant lesions.

Data from this study increase the oral surgeon’s index of suspicion when treating patients with erupted and partially erupted lower third molars and encourage clinicians to carefully follow up on radiolucent DALM lesions. Moreover, it would be useful to conduct a study that aims to evaluate the percentage of DALM radiolucency (< 2.5 mm) evolving into osteolytic lesions of the jaw bones know of such, order to better understand if the preventive extraction of these teeth is recommended in these clinical circumstances.

### Fi-index tool

This manuscript has been checked with the Fi-index tool and obtained a score of 0.55 for the first author only on the date 21/02/2023 according to SCOPUS®. The fi-index tool aims to ensure the quality of the reference list and limit any auto-citations [20, 21].

## Data Availability

Data are available on request to the Corresponding author.
